# A case of ossifying fibroma of the frontal sinus

**DOI:** 10.3892/etm.2013.1002

**Published:** 2013-03-12

**Authors:** NA SUN, WEI-HUA XU, LU-HONG CAO, XIAO-YAN ZHAO, JING-FEI ZHANG, JIA LI, WEN-PING LI, GUANG-BIN SUN

**Affiliations:** 1 Department of Otolaryngology, Gongli Hospital, Pudong New Area, Shanghai 200135;; 2 Department of Otolaryngology, The Affiliated Hospital of Ningxia Medical University, Yinchuan, Ningxia 750004;; 3 Department of Pediatrics, People’s Hospital of Pudong New Area, Shanghai 201200;; 4 Department of Otolaryngology, Nanhui District Central Hospital of Shanghai, Shanghai 201300, P.R. China

**Keywords:** ossifying fibroma, benign tumors, nasal cavity, paranasal sinus

## Abstract

Ossifying fibroma is a rare benign tumor of the nasal cavity and the paranasal sinus, and is easily misdiagnosed. In the present study, we report the clinical data in the case of a 46-year-old female patient, treated due to 5-day forehead swelling accompanied by dizziness. CT examination revealed dilation of the right frontal sinus, bone wall integration, dense masses in the cavity, multiple punctate calcification foci internally and no nasal obstruction, nasal mucus or epistaxis. After hospitalization, a right frontal sinus fenestration and tumor resection plus nasofrontal duct reconstruction combined with nasal endoscopic frontal recess open surgery was conducted under general anesthesia. Following the tumor resection, the frontal sinus bone lamella was reset and fixed with a titanium bone fixation set. The postoperative pathological diagnosis was of ossifying fibroma. At the postoperative 5-year follow-up there was no tumor recurrence and nasal endoscopy revealed an unobstructed nasofrontal duct opening.

## Introduction

Ossifying fibroma is a rare benign tumor of the nasal cavity and paranasal sinus, and is easily misdiagnosed. Since the initiation of nasal endoscopies in the Department of Otolaryngology (Gongli Hospital, Shanghai, China) we have treated two cases of ossifying fibroma in the nasal cavity and paranasal sinus, confirmed by pathology, over a period of 5 years (2003–2008). Of the two, only one case has complete clinical data. In the current study we summarize the clinical and pathological data and follow-up results of this case. The study was approved by the ethics committee of Gongli Hospital. Informed consent was obtained from the patient and the patient’s family.

## Case report

A 46-year-old female patient was treated due to 5 days of forehead swelling accompanied by dizziness. The patient received a head CT in the Neurology Department, which revealed a right frontal sinus lesion (possibly caused by inflammation due to a fungal infection), and was subsequently transferred to the Department of Otolaryngology, Gongli Hospital for treatment. The patient was hospitalized on April 17, 2007. From the onset, she had no vertigo, apparent nasal obstruction, nasal mucus, epistaxis, hyposmia or headache. Furthermore, she denied any history of trauma to the head or face.

**Figure 1:**
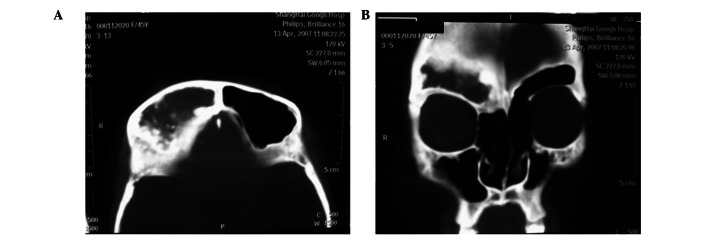
Head CT shows that the right frontal sinus was dilated, the bone wall was integrated, there were dense masses in the cavity and multiple punctate calcification foci internally, the boundary was clear, and there were surrounding bone-like density shadows. (A) Horizontal position. (B) Coronal position.

At the admission examination, the systemic signs were good and the external nasal shape was normal. Right middle meatus mucosal edema was visible under the anterior rhinoscope but there were no newplasm or purulent secretions in the nasal cavity. The right part of the forehead bulged slightly, the skin was normal, there was no local tenderness or fluctuation, the position of the right eyeball was slightly lower than that of the left eyeball but eye movements, visual acuity and visual field were all normal. CT scans revealed that the right frontal sinus had dilated, the bone wall was integrated, dense masses were present inside the cavity and there were multiple punctate calcification foci internally. Furthermore, the boundary was clear, there were shadows of bone-like density, the superior wall of the eye socket had moved down under stress, and sclerotin was attenuated due to mild absorption (Fig. 1).

**Figure 2:**
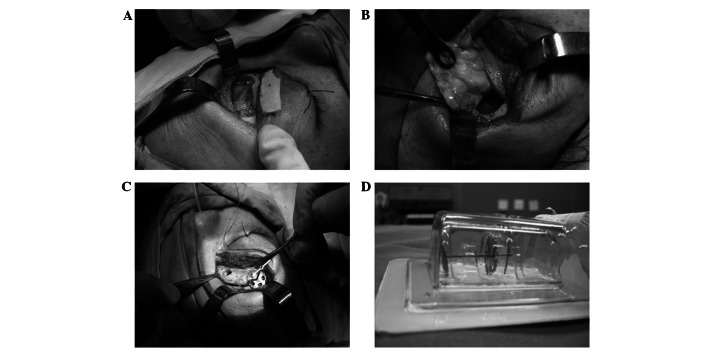
Surgical procedure. (A) Anterior wall fenestration of the frontal sinus. (B) The surgical cavity was filled with grey-white block-like masses and sand-like tissues. The tumor had capsule and like bone, and the boundary was clear. Complete dissection was conducted. (C) The frontal sinus bone lamella was reset and fixed with a titanium bone fixation set. (D) Titanium bone fixation set for use in the surgery.

**Figure 3:**
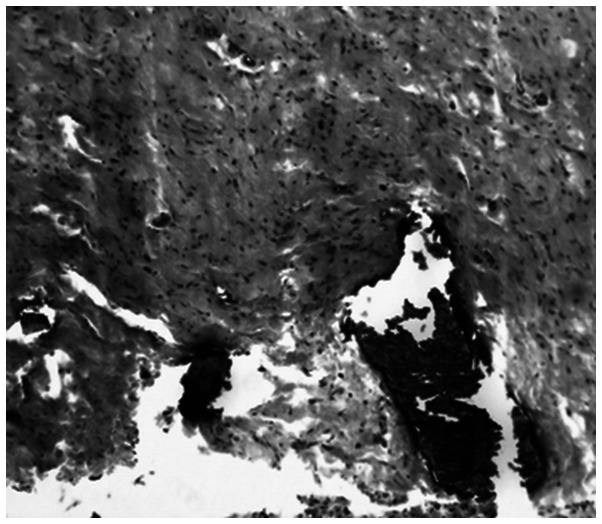
Ossifying fibroma H&E staining (magnification, ×200).

On the 4th day of hospitalization, a right frontal sinus fenestration and tumor resection plus nasofrontal duct reconstruction combined with nasal endoscopic frontal recess open surgery was performed under general anesthesia. During the surgery, a curved incision was made 0.5 cm from the right side of the inner canthus outwards to the interior superciliary arch, and a bone window of ∼4×2 cm was prepared at the anterior wall of the frontal sinus in order to expose the frontal sinus cavity. It was observed that the sinus was filled with a solid mass, ∼4.5×4×0.6 cm in size (grey-white block mass) and sand-like tissues. The tumor had capsule and like bone, and the boundaries were clear. Following complete dissection, the surrounding bone wall presented stalactite-like changes in appearance, and the surface was rough. At the frontal sinus opening, there was a bony nodular prominence (0.5×1 cm), the opening of the frontal sinus at the sinus cavity surface was completely affected by bony atresia. An electric drill was used to grind the bony prominence in the sinus cavity, and the anterior ethmoid sinus and frontal recess were opened using a nasal endoscope. From the bottom wall of the frontal sinus, sclerotin was gradually ground downwards along the normal nasofrontal duct. Subsequently, the nasofrontal duct was reconstructed. A silicone tube was placed into the frontal sinus, led out from the anterior naris though the nasal cavity and fixed. The frontal sinus bone lamella was reset and fixed with a titanium bone fixation set, and the frontal incision was sutured layer by layer. A pressure dressing was applied and the anterior ethmoid sinus cavity was filled with calcium alginate gauze (Fig. 2). There was little intraoperative bleeding and no eyelid swelling or eyeball movement disorder following the surgery. On the 3rd postoperative day, the nasal cavity gauze was removed. On the 7th postoperative day, the suture was removed and the frontal sinus drainage tube was retained. Additionally, the frontal sinus was rinsed daily. The postoperative pathological diagnosis was ossifying fibroma (Fig. 3). On the 10th postoperative day, the patient left hospital, and the forehead swelling and dizziness had disappeared.

**Figure 4:**
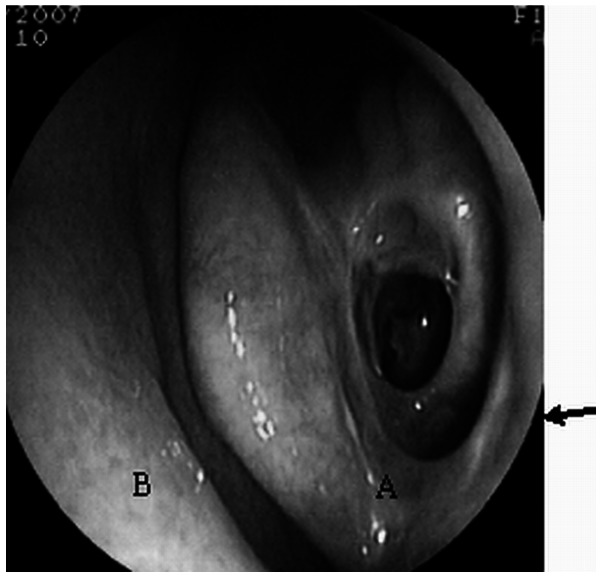
Electron nasopharyngolaryngoscopy at the postoperative 1st year showed that the right nasofrontal duct opening was unobstructed. A, middle nasal concha; B, nasal septum. The arrow indicates the nasofrontal duct opening).

**Figure 5:**
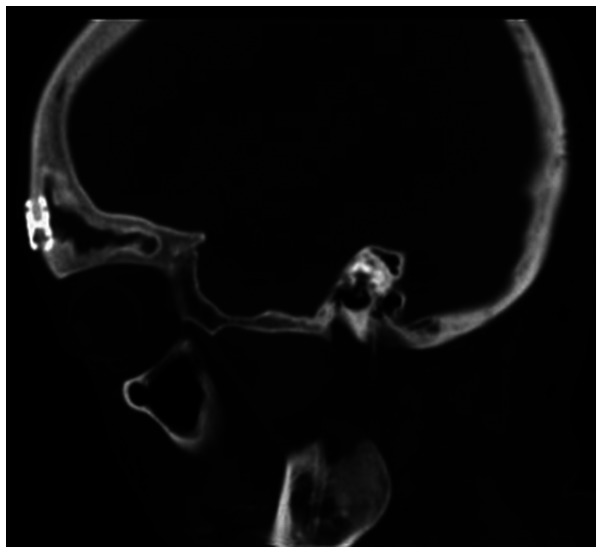
Frontal sinus sagittal view CT at 1 year postsurgery demonstrated that the titanium bone fixation set was in place and that there was no space-occupying lesion in the frontal sinus cavity.

After half a month, reexamination revealed that the frontal sinus drainage tube in the nasal cavity was unobstructed and the frontal sinus was rinsed. After one month, the frontal sinus drainage tube was removed, and nasal endoscopy was performed, revealing that the nasofrontal duct opening was unobstructed. At the 5-year postoperative follow up, there was no tumor recurrence. Nasal endoscopy 1 year postoperatively revealed an unobstructed nasofrontal duct opening (Fig. 4). The 1 year postoperative paranasal sinus CT is shown in Fig. 5.

## Discussion

Since the first case of ossifying fibroma was reported in 1872, there was a period of time when ossifying fibroma and fibrous dysplasia of bone were considered to be the same disease (1). Following a long period of observation and study, they are now considered to be two different diseases. Ossifying fibroma is a bone tissue-derived benign tumor, whereas fibrous dysplasia is a hyperplastic bone lesion caused by bone mesenchymal dysplasia (2).

Ossifying fibroma is a rare bone tissue-derived benign tumor occurring in the craniofacial bone. Of all cases, 70% occur in the maxilla and mandible (particularly in the maxilla). The frontal bone, ethmoid bone and sphenoid bone may all be affected. Occasionally, the occipital and temporal bones have been affected (2). Ossifying fibroma occurs mainly in childhood and adolescence, occasionally in patients aged 20–40 years old, and rarely in patients >40 years old. Triantafillidou *et al* hypothesized that the lesions in adult patients had developed during adolescence (3). The cause of this disease is unknown. Currently, there are three theories: the dysplasia theory, the trauma theory and the tumor theory. Current thinking tends towards the tumor theory, which postulates that the disease is a bone tissue-derived new generation product (2) and trauma is only a precipitating factor of this disease.

The clinical symptoms of this disease have no marked specificity, and early detection is difficult. The patient in this study was 46 years old, and the lesion had possibly occurred earlier. Due to the lack of clear clinical symptoms, she had not visited a doctor. Her clinical manifestations were mainly dizziness, local swelling and eyeball dislocation and she had no nasal symptoms. Therefore, the diagnosis was missed by doctors in the otorhinolaryngology department. The case was initially diagnosed in the neurology department due to the dizziness symptom.

The diagnosis of the case was dependent on paranasal sinus CT and pathological examination. As a routine means of examination, paranasal sinus CT assists diagnosis and defines the lesion range for preparing a surgical intervention, and it should act as a routine presurgical examination. CT scanning images of the nasal cavity and paranasal sinus present round or oval high-density shadows, where the density is uniform. If there is bleeding or a cystic lesion in the tumor, partial areas present as low density shadows, there are uneven-thickness bone shells on the surface and the boundary is clear. Additionally, pathological examination is critical for a definitive diagnosis. Pathological examination reveals a clear boundary, a fibro-osseous lesion and sand-like material distributed in the fibrous stroma. These are always the main features of ossifying fibroma. A preoperative biopsy is difficult to perform, as it is essential to cut open the mucus in order to take out the central lesion of the tumor. However, this method may easily induce the enclosed mass to bleed. It is recommended that a biopsy is not conducted prior to surgery. Furthermore, it is necessary to differentiate ossifying fibroma from fibrous dysplasia of the bone. A CT of the latter generally shows a more even mass density and the boundary is not clear, giving an appearance more similar to that of frosted glass. Pathological characteristics include irregular bone trabeculae, disorganized and crisscrossed collagen fibers and a large number of osteoclasts in the bone matrix, and no definite boundary between the lesion and the surrounding tissues. Some investigators are of the opinion that histochemistry and immunohistochemistry may aide the identification of these characteristics.

Ossifying fibroma in the nasal cavity and paranasal sinus is a benign tumor histologically, but in the clinic it has invasive characteristics. It usually invades the eye socket, the base of the skulland calvarium to induce symptoms in the eye and brain, and its malignancy rate is 0.4–0.5% (4). Furthermore, it is mainly induced due to repetitive surgery or chemotherapy. Therefore, there is only one effective treatment method: an early surgery for complete tumor resection. There are many approaches to the surgery, and the most suitable approach is generally determined according to the lesion range (5). For lesions that occur only in the maxillary sinus, the selection of the Caldwell-Luc approach for conducting a maxillary sinus radical operation is appropriate. For lesions that occur in the anterior ethmoid sinus, frontal sinus and sphenoid sinus, the selection of a lateral rhinotomy or an extended lateral rhinotomy is appropriate. For cases with a wide lesion range and with tumor invasion of the base of the skulland other important sites, it is feasible to use a combined craniofacial approach for the complete resection of the tumor. Some investigators believe that as the tumor invasion site is critical and the surgical risk is high, it is necessary to conduct a partial resection of the tumor to ensure the function of vital organs (6).

The previous literature, as well as the findings from the current case, suggest that the prognosis of this case is good. There was no tumor recurrence following the complete resection of the tumor. Recurrence mostly occurs due to incomplete surgery, and following recurrence, the only solution is to perform a complete resection of the tumor again. Radiotherapy and chemotherapy are invalid for this disease, as radiotherapy may promote a malignant transformation resulting in the formation of an osteosarcoma (6).
